# Effect of the Mechanical Properties of Soft Counter-Faces on the Adhesive Capacity of Mushroom-Shaped Biomimetic Microstructures

**DOI:** 10.3390/biomimetics8030327

**Published:** 2023-07-24

**Authors:** May Gonen, Haytam Kasem

**Affiliations:** 1 Faculty of Mechanical Engineering, The Technion-Israel Institute of Technology, Haifa 32000, Israel; 2 Department of Mechanical Engineering, Azrieli College of Engineering, Jerusalem 9103501, Israel

**Keywords:** biomimetic, mushroom-shaped microstructure, adhesion, substrate, mechanical proprieties

## Abstract

The effects of mechanical properties and contact environment conditions on the adhesiveness of the biomimetic adhesive mushroom-shaped micro-structure have been experimentally investigated. The idea is based on the adhesive micro-structures and surfaces inspired by nature after observing the abilities of some animals. Applications are proposed in various fields of engineering and technology. However, to enable unconventional uses of these biomimetic adhesion surfaces, such as in the biomedical field, it is necessary to adjust and optimize their tribological properties (friction, adhesion, and peeling strength) in contact with soft substrates that can simulate the mechanical features of biological tissues. Our work explores the effect of the combinations of the various parameters on the strength of adhesion. Under dry contact conditions, soft counter-faces lead to lower adhesion than hard counter-faces, whereas under wet conditions, soft counter-faces lead to higher adhesion than harder counter-faces.

## 1. Introduction

Over the few last decades, the field of adhesive sciences has evolved due to the growing need for reversible and rapid adhesive systems in various fields of technology [1,2,3]. These developments were inspired by the biological adhesive systems found in several species of insects, reptiles, and spiders, which have developed unique biological attachment systems during their natural evolution. These systems allow them to grip and run on the wide range of horizontal, vertical, rough, and smooth surfaces that they encounter in their living environments [4,5,6]. Systems based on permanent or long-term adhesion mainly rely on mushroom-shaped micro-structures, while systems involved in short-term temporary adhesion mainly rely on the spatula shape of individual contacts [1].

One of the dry biomimetic adhesives developed for real use is based on mushroom-shaped contact elements [4]. Inspired by the sticky hairs found in male beetles from the Chrysomelidae family, this microstructure does not present a hierarchical geometry like that found in the biological attachment system of the gecko; rather, it is simply a surface covered with mushroom-shaped microstructures. This attachment system is suitable for creating a long-term passive adhesive force on smooth substrates with almost no pre-load. The potential of these biomimetic adhesives was first verified using a robot-type device walking on smooth vertical surfaces that used this microstructure [7]. The tribological performances of mushroom-shaped adhesive microstructures attracted the attention of many researchers during the last decade. Research was conducted to investigate their various properties and the influence of different operational parameters. Pre-load and contamination have been studied by Gorb et al. [4]. They compared the adhesive properties of a biomimetic mushroom-shaped fibrillar microstructure to that of a control flat surface that was made of the same material and operated under the same operational conditions, and they proved that the adhesive features of the structured surface were more than twice as effective as those of thee flat surface.

The influence of pre-load on the adhesion was studied by Varenberg et al. [8]. They showed that the mushroom-shaped geometry of fibrillar contact elements was responsible for a stable adhesive attachment. This type of contact element promotes the fast and simple generation of reliable adhesion. The mushroom-shaped geometry seems to transform fibrillar contact elements into passive suction devices and makes them tolerant to overload, thus enhancing their robustness and stability [9]. Additional parameters have been studied, such as the rule of hierarchy. These works show that the adhesion enhancements are the result of increased surface conformation [10]. In addition, it is reported that ambient pressure and suction may contribute up to 10% of the pull-off force measured on the structured surfaces at high velocities [9], while oil lubrication (wet adhesion) involves both capillarity- and viscosity-dependent forces [11]. As for performance under different contact conditions, reversible adhesion has previously been achieved using a mushroom-shaped microstructure, which is inspired by the beetle’s microbial structure, submerged underwater [12]. Surfaces with a defined structure have a 25% increase in adhesion when immersed in water compared to a dry surface. Adhesion of a mushroom-shaped microstructure via underwater contact is 20 times more effective than that of a flat surface made of the same material. The Van der Waals interaction that creates adherence is greatly enhanced via the suction effect that occurs in underwater interaction. The resulting higher adherence of the substance encourages possible applications in biomedical technologies, as well as a variety of applications in which mushroom-shaped microstructures are submerged in fluid environments. Thus, it is important to note that in a wide variety of engineering applications, biomimetic mushroom-shaped adhesive microstructures are usually used in dry contact, whereas natural methods usually contains fluid [13]. 

In fields related to engineering, such as machines and robots, the tribological performances (adhesion, friction, and peeling strength) of biomimetic adhesive microstructures are often evaluated using smooth and hard counter-faces (in general glass). However, as shown by research into biological or medical applications, it is becoming necessary to ensure that counter-faces made of soft materials are as close as possible to the mechanical properties of biological tissues. In the light of the above issues, the present study aims to experimentally investigate the influence of the mechanical properties of different soft and hard counter-faces on the adhesive strength of biomimetic mushroom-shaped micro-structures. Their adhesive capacity will also be evaluated under different contact conditions. The inspiration for modeling our soft counter-faces emanated from a study that investigated the effect of surface micro-structures on the friction and lubrication properties of the tongue-based tribological system model [14]. Given that the mushroom-shaped contact elements are commonly developed to achieve passive long-term adhesion, while they often fail to generate friction resistance, in this study, only their adhesive properties will be investigated.

## 2. Materials and Methods

### 2.1. Mushroom-Shaped Microstructure and Flat Reference Samples

In this study, the mushroom-shaped microstructure tape used was manufactured by Gottlieb binder GmbH (Holzgerlingen, Germany) [15]. The manufacturing process consisted of pouring two-compound polymerizing poly(vinylsiloxane) (PVS; Coltene Whaledent AG, Altsatten, Switzerland) to 0.3-millmeter thick cast tape with Young’s modulus at around 3 MPa [16], and the tape that contained the microstructures was then released from the negative template. The use of such a soft elastomer helped us to obtain very compliant structures that increase adhesive performance. Indeed, the compliant structures barely store elastic repulsive energy and, therefore, easily follow the roughness of the counter-face with which they are in contact, thus increasing the intimate contact area and the resulting adhesive forces. The obtained mushroom-shaped microstructure consisted of hexagonally packed pillars of about 100 µm in height, bearing terminal contact plates of about 40 µm in diameter, and an areal density of the terminal contact plates of around 40%, according to our calculations. The backside of the cast micro-structured tape was used as a smooth reference surface, as it was made of the same material.

**Figure 1:**
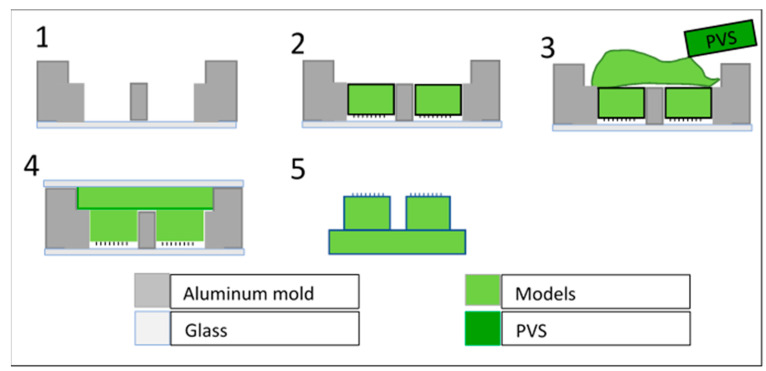
Schematic description of the process of integration of the small biomimetic cylinders into a single model. (1) the specific mold positioned on a smooth and clean glass; (2) small cylinders containing the mushroom elements inserted inside the specific mold with textures facing the glass; (3) a small quantity of PVS gently poured over the backside, which once solidified fixes their position together; (4) a cover glass used to remove extra PVS and to unify the shape and thickness of the final sample; (5) release of the final combined sample after PVS solidification.

To prepare samples to fit the customized adhesion test-rig used in this study, the adopted concept placed six small cylinders (∅ 2 mm and 1 mm in height) on the same sample, while all cylinders faces, whether they had either mushroom-shaped or smooth flat faces, were aligned on the same plane. To complete this step, the process reported in reference [17] was used. A specific aluminum template was manufactured via a CNC process. The mold contained a socket with six round holes in the bottom, which were arranged symmetrically to achieve, as far as possible, an equal load distribution, which is a necessary condition in tribological characterization. The process consisted of placing the aluminum mold onto a glass panel, before inserting the six cylindrical models into the holes of the biomimetic microstructures, meaning that the tested side was in contact with the glass to enable flattening, thus verifying that the cylinders were aligned in the same plane. Next, a PVS fixative elastomer was placed on top of the aluminum mold to unite the six different cylinders into one model. A second flat glass was used to flatten the PVS to achieve a uniform thickness of the final model. Finally, after polymerization, the resulting model was released from the aluminum mold (Figure 1).

**Figure 2:**
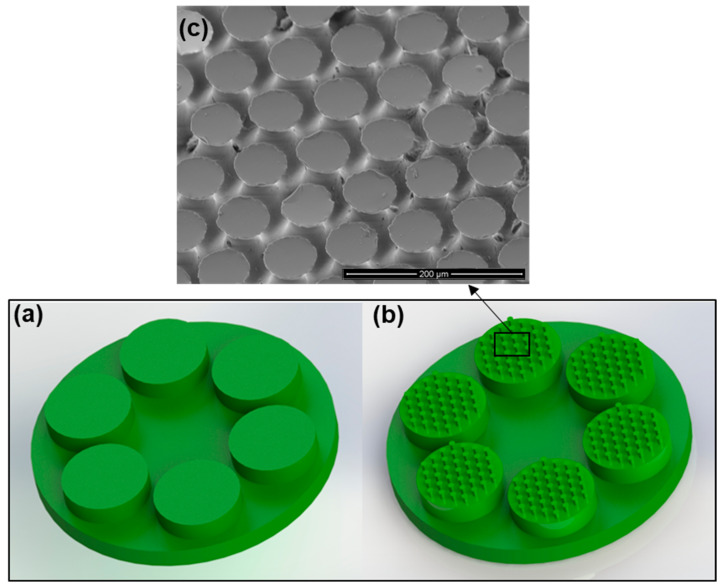
Illustration of the (**a**) flat control sample, (**b**) mushroom-shaped biomimetic microstructure sample, and (**c**) SEM image of the mushroom-shaped pillars.

Following the preparation process described above, two different samples were prepared and tested: a flat reference sample in which all six sub-contract points were cylinders with flat smooth surfaces (see Figure 2a), and a mushroom-shaped microstructure sample in which all six sub-contract points were cylinders with mushroom-shaped microstructures (see Figure 2b). It is important to note that all cylinders (micro-mushroom-shaped and flat) were randomly placed inside of the aluminum mold due to their isotropic property.

### 2.2. Counterface (Substrate)

In contrast to previous works, in which only one hard material was used for the counter-face, general glass, or Epoxy [18,19,20], in the present study, the adhesion experiments were performed on three different counter-faces, which were duplicated from the same smooth surface (Microscope slide) using three different materials that had different mechanical properties, i.e., PVS, SILFLO©, and Epoxy used in this study as a reference counter-face material. All counter-face specimens (25 × 20 × 1 mm^3^ in size) were cast via replicating the same surface (Microscope slide) using a two-step molding technique [21]. PVS (Poly-vinyl siloxane), which is an addition–reaction silicone elastomer, has a Young’s modulus of E_P_ = 3.12 MPa once polymerized [13]. SILFLO© is a brand of silicone impression material that consists of a base and catalyst in a putty consistency. It is a soft material that has a Young’s modulus of about Es = 1.5 MPa. This material is mainly used to simulate mechanical proprieties of biological tissues, such as the tongue [14]. Epoxy is a hard resin used to manufacture adhesives, coatings, and other products and materials. It has a Young’s modulus about E_E_ = 3.1 GPa [8]. Epoxy is also used to cast different counter-faces (substrates) in previous studies [19,20]. 

**Figure 3:**
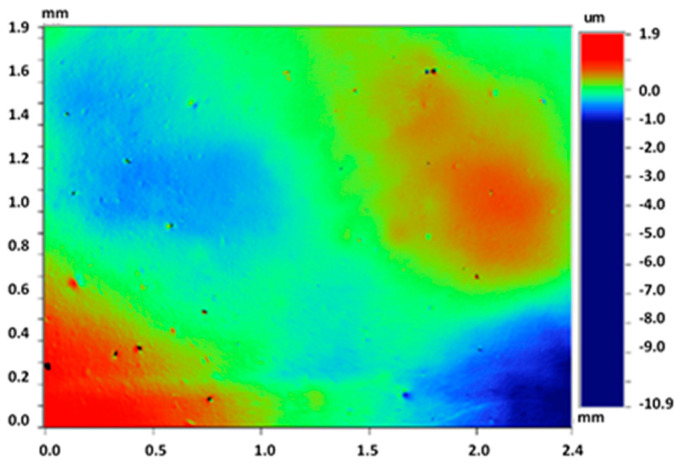
3D optical profilometer image of the tested counter-face.

These counter-face specimens were fully characterized using 3D optical profilometer (Figure 3) Wyko NT1100 (Veeco, Tucson, AZ, USA). The counter-faces were examined three times using different areas on the surfaces. The main roughness parameters measured, for which average values are shown in Table 1, are as follows:

*Ra*—the average roughness calculated over the entire measured array;

*Rq*—the root-mean-squared roughness calculated over the entire measured array;

*Rz*—the average of the ten greatest peak-to-valley separations;

*Rpk*—reduced peak height, i.e., the top portion of the surface that can be worn away during the run-in period;

*Rvk*—reduced valley depth, i.e., the lowest portion of the surface that might retain lubricant during wet contact. 

The contact wettability angle was measured via water contact angle measurement. The measurements were conducted with a droplet of double-distilled water (DDW) using an Easy Drop contact angle goniometer (FM40Mk2, Krüss GmbH, Hamburg, Germany) at room temperature and ambient humidity. The contact wettability angle characterized the properties of the surfaces in terms of hydrophilicity (θ < 90°) or hydrophobicity (θ > 90°).

We noted that the sample replicated in this study was obtained in a previous work [19] (Microscope slide), while the roughness parameters obtained are very similar and are within the measurement error range. Therefore, it can be concluded that the samples were properly prepared in the present study.

### 2.3. Experimental Procedure

Adhesion experiments were performed via a customized tribometer that was developed at the Laboratory of Tribology and Microstructures of the Azrieli College of Engineering, Jerusalem (JCE). A full description of the used device is given in [17]. Based on a moving horizontal counter-face, this tribometer allowed us to evaluate the tribological properties (friction, adhesion, and peeling) of different materials, including textured surfaces, under dry or wet contact conditions according to needs. Using this tribometer, the drive unit consisted of three translation stages (two motorized and one manual) to adjust the contact location and apply the loads between the friction pair components. The measurement unit consisted of two load cells (FUTEK’s FSH00092-LSB200) used to measure force variations at a high resolution (0.1 mN) in both the normal and tangential directions. The operating and control software were written in a LabVIEW environment. The measurements were sampled via a multifunctional data acquisition board Lab-PC- NI USB-6211 (National Instruments Co., Austin, TX, USA) and processed using the LabVIEW 2017 software package (National Instruments Corporation, 11500 N. Mopac Expressway, Austin, TX, 78759, USA). The current study used a passive self-aligning system to ensure full flat-on-flat contact between the mating surfaces during the adhesion experiments. 

**Figure 4:**
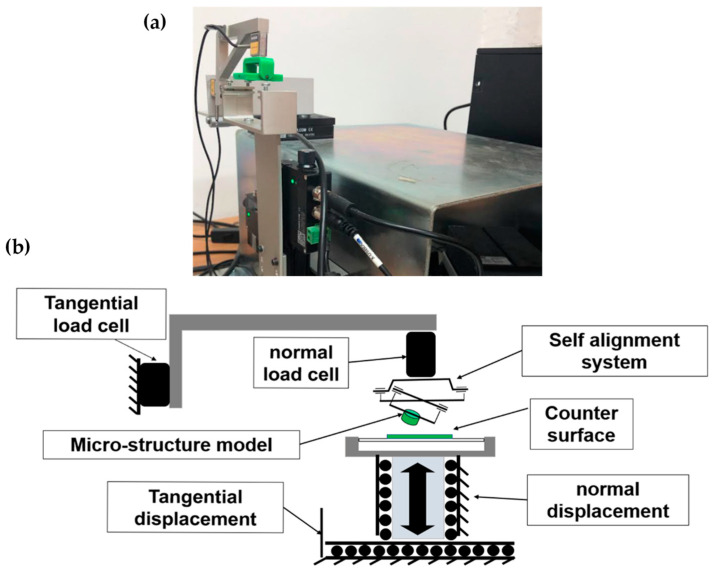
(**a**) General view and (**b**) schematic illustration of the customized tribometer.

The samples, i.e., flat sample (FS) or mushroom-shaped microstructure sample (MSMS), were mounted on the holder and connected to the self-alignment system. Next, the selected counter-face specimen, which was already glued to a microscope slide glass, was mounted on the moving holder attached to the translating stage. The fixation screws were then reinforced to prevent any unwanted movement (see Figure 4).

Once the samples were mounted, the measurement and self-alignment systems were calibrated by resetting the load cells to eliminate the effect of mass gravity. It is important to note that the same calibration was performed after each sample replacement. Adhesion tests were conducted as follows: The counter-face specimen was brought into contact with the patterned microstructure samples at a pre-defined speed, leading to a gradual increase in the normal load P until the pre-defined value was reached. Next, the translation stage was withdrawn in the normal opposite direction at a pre-defined constant velocity, while the load cell measured the generated pull-off force. The maximal adhesion force at the separation point was recorded for each test. Each sample or configuration was tested four times, from which tests the average value of the maximal adhesion force, as well as the standard deviation, was calculated.

The adhesion strength presented in the graphs of the experimental results was computed by dividing the measured adhesion force by the total contact area of the sample (6 small cylinders, see Figure 2). Next, the obtained value was normalized over the nominal aspect ratio of contact surface *η* (Equation (1)). This value was equal to 1 for the smooth control reference model and 0.4 for the mushroom-shaped microstructure model.

(1) $$\eta =\frac{S}{∆S}\cdot 100\;[\%]$$

where *S* is the relative area of the mushroom, and *∆S* is the total equilateral area.

All experiments were performed under the same ambient condition at a room temperature of 23 °C ± 1 °C and a relative humidity of 45% ± 5%.

### 2.4. Contact Environment

**Figure 5:**
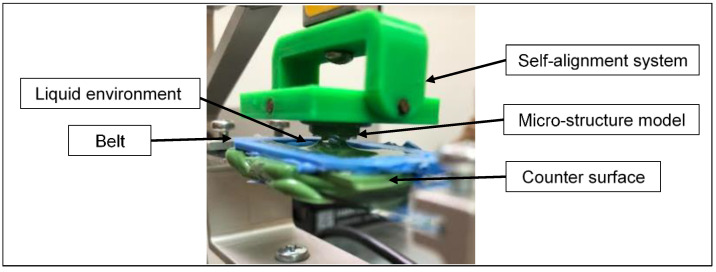
Glass slide microscope with a PVS belt during an adhesion experiment under contact when submerged with glycerol.

In the present study, the adhesion experiments were performed under three different environment contact conditions, i.e., dry (in the air), distilled water, and glycerol. To retain the liquid on the counter-face (substrate) for the experiments performed under distilled water and glycerol, a PVS belt of 1 mm height was glued onto the contour of the counter-face (see Figure 5).

### 2.5. Tests Operational Conditions

Each model was tested by applying normal loads of 100, 200, 300, 500, 700 and 1000 mN to cover the load range inferred in previous studies [19,20] (giving nominal contact pressures on the mushroom-shaped microstructure: 0.013 to 0.13 MPa). The loading and unloading speed was 0.5 mm/s.

## 3. Results and Discussion

**Figure 6:**
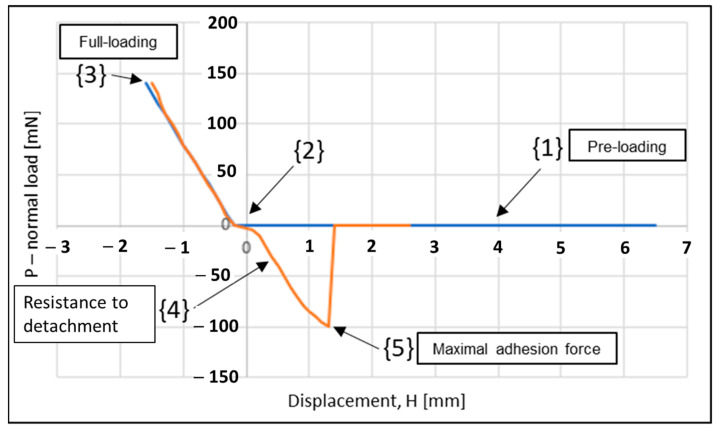
Force–distance curve showing the typical behavior during adhesion test. Loading (blue line) and unloading (orange line).

Figure 6 presents the typical behavior of an adhesive contact, which occurs when the normal load is displayed as a function of the vertical displacement Hmm (distance between the model and the substrate “counterface”). This behavior can be divided into five characteristic stages: In stage (1), models approach the opposite models before making contact with each other, and in stage (2), the models (smooth control reference model or mushroom-shaped microstructure model) come into contact with the counter-face. In step (3), the system is loaded until it reaches the desired value of the normal pre-load, and in step (4), the resistance to detachment is measured as a function of displacement in the opposite direction at a pre-determined separation speed. When there is no adhesion, disconnection occurs almost immediately. However, when the contact is adhesive, disconnection does not occur immediately. The force continues to decline in the negative stage due to resistance to disconnection. Full disconnection occurs at point P_a,max_ (5), which corresponds to the maximum adhesion force measured for each test. Finally, the system stabilizes after slight fluctuations.

As mentioned above, in this study, we investigated the influence of counter-face material under different environmental conditions.

### 3.1. Dry Contact Condition

**Figure 7:**
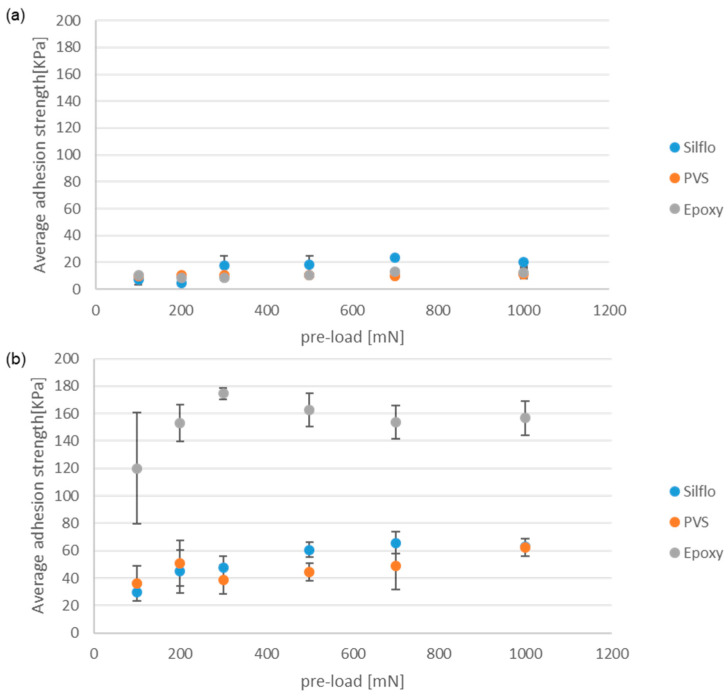
The average values of the maximal adhesion force under dry contact conditions. (**a**) The smooth control reference model and (**b**) the mushroom-shaped microstructures. All counter-faces are replicated using the same microscope slide.

Figure 7 displays the average values of the maximal adhesion strength obtained using the three counter-face materials (epoxy, PVS, and SILFLO©) that have almost the same surface roughness (Ra around 0.1–0.5 µm, replicated from a microscope slide) under dry contact conditions. The maximal adhesion strength is displayed as a function of the applied normal pre-load. Data presented in (a) were obtained via a smooth control reference model, while data presented in (b) were obtained via a mushroom-shaped microstructure model tested under the same operational conditions.

The performance of mushroom-shaped microstructures can be seen in Figure 7b. The trend lines suggest that the maximum adhesion force appears to be unaffected by the value of the initial normal pre-load. This behavior has already been reported in the literature [20]. Indeed, a certain minimum pre-load value is required to form the maximum contact area between the mushroom-shaped microstructures and the opposite counter-face, beyond which no additional contact area can be achieved. As for the current results, it is likely that the minimum pre-load applied is higher than the requested minimum preload. Under dry contact conditions, the hard epoxy counter-face gives the highest adhesion strength, while the softer materials (PVS and SILFLO©) give smaller adhesion strengths that are close to each other, with a slight advantage for SILFLO©. These results tend to highlight that, at least in the case of dry contact conditions, the high mechanical properties of the substrate (counter-face) do not affect negatively the adhesion force when in contact with biomimetic adhesive elements such as mushroom-shaped microstructures. This result can be explained by the stress concentration distribution on each mushroom cap [21]. When separating a soft micro-mushroom element from a softer counter-face, stress concentration occurs at the edge of the mushroom cap, leading to initial detachment from the side toward the center of the mushroom. This behavior leads to relatively fast detachment, reducing the measured adhesive force [21]. This behavior can be approximated using the model of mushroom-shaped pillar with a thick plate described in [22], in which the interfacial stress singularity appears at the cap’s edge, probably due to the diminution of the stiffness ratio between the mushroom caps and the counter-face. In contrast, when separating a soft mushroom cap model with optimal plate thickness from a rigid counter-face, the stress concentration occurs in the center of the mushroom [23]. In this case, the initial detachment between the mushroom caps and the rigid counter-face begins at the center of the pillar and propagates under the shape of circumferential peeling, progressively increasing the peeling line which, when coupled with the resulting artificial suction effect, contributes positively to increasing adhesion force [12]. Figure 7a is related to the reference smooth control model. For all three materials, the adhesion strength is lower than that of the mushroom samples (Figure 7b). SILFLO©, however, gives slightly higher adhesion than the two other materials, especially when the applied normal pre-load is higher than 300 mN. In Figure 7, it can be seen that under dry conditions, there is a very limited influence of the pre-load, as was previously reported in [4].

### 3.2. Wet Contact Environment—Distilled Water

**Figure 8:**
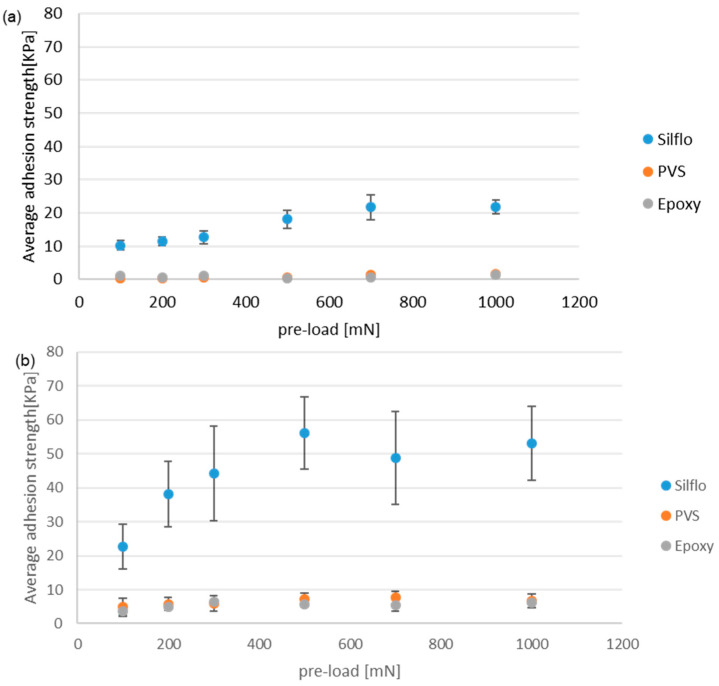
Average values of the maximal adhesion strength as a function of the applied normal pre-load under wet contact conditions with distilled water for two models: (**a**) a smooth control reference model and (**b**) mushroom-shaped microstructures. All counter-faces are replicated using a microscope slide.

Figure 8 displays the average values of the maximal adhesion strength for (a) the smooth reference and (b) mushroom-shaped microstructure samples under water-wet conditions. The maximum adhesion strength is displayed as a function of the applied normal pre-load for the three counter-face materials (Epoxy, PVS, and SILFLO©), which have the same surface roughness. The contact was completely submerged within distilled water during the adhesion test (see illustration in Figure 5).

When submerged with distilled water, the hard Epoxy counter-face shows almost non-adhesive behavior relative to the smooth reference (a), while there is a very low value for the textured sample (b). However, concerning soft material, SILFLO© presents the highest adhesion (on average 8 to 10 times higher than those of the other two materials). Humidity-related effects on adhesion can be explained based on the capillary forces due to the formation of liquid bridges [24]. Hence, the low elasticity modulus, when combined with possible capillarity forces, seems to be the cause of its high adhesion capacity within distilled water. The low elasticity modules of the SILFLO© counter-face, when combined with the high flexibility of mushroom caps under water, might accentuate the effect of artificial suction, as reported in [8,25,26], hence the increasing adhesion force. The slightly higher elasticity modulus and greater hydrophobicity of PVS than SILFLO© seems to be the cause of its lower adhesion strength.

### 3.3. Wet Contact Environment—Glycerol

**Figure 9:**
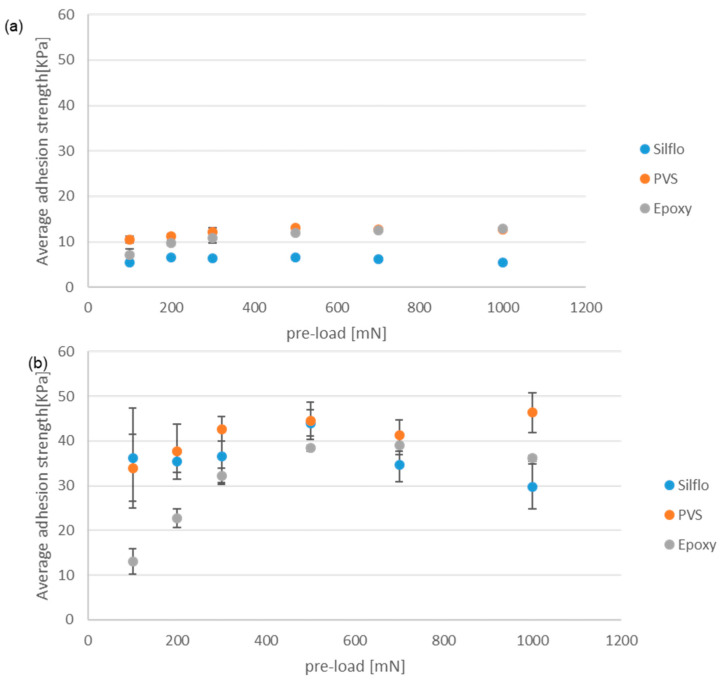
Average value of the maximal adhesion strength as a function of the applied normal pre-load under wet contact conditions with glycerol for two models: (**a**) a smooth control reference model and (**b**) a mushroom-shaped microstructure. All counter-faces are replicated using a microscope slide.

Figure 9 displays the average values of the maximal adhesion strength for (a) smooth reference and (b) mushroom-shaped microstructure samples tested under Glycerol wet condition (contact submerged with glycerol during the adhesion test). The maximum adhesion strength is displayed as a function of the applied normal pre-load for the three counter-face materials (epoxy, PVS, and SILFLO©), which have the same surface roughness.

In the case of mushroom-shaped microstructure sample (b), when tested under small pre-loads, the hard epoxy counter-face generates an adhesion strength smaller than those of the two other soft materials (PVS and SILFLO©). This behavior can be explained based on the fact that under small pre-loads, the hard epoxy does not deform enough to generate sufficient contact surface with the mating mushroom-shaped micro-structures. It is also possible that glycerol contained air bubbles that were retained between the mating surfaces under small pre-loads. In contrast, the PVS and SILFLO© counter-faces deformed more noticeably under the same normal pre-load, which is something that, along with the deformation of the patterned mushroom-shaped microstructure, contributes to the increase in the real contact area, leading to the generation of higher adhesion force. While PVS gives better results (highest adhesion), the difference with SILFLO© is insignificant. SILFLO© still presents the best enhancement ratio between the smooth reference and mushroom-shaped microstructure samples.

It is important to note that the present work does not take in consideration the influence of the detachment velocity, which can affect the behavior of the interface. In another recently published work [27], it was shown that the adhesion properties of biomimetic mushroom- and spatula-like elements were affected by the detachment velocity, and three different regimes were reported: (i) a quasi-static range, in which no clear dependent was obtained; (ii) an intermediate range, in which the maximum adhesion force at detachment increased in line with the detachment velocity; and (iii) an upper limit, which represents a velocity beyond which the pull-off force no longer depends on the detachment velocity.

## 4. Conclusions

The present work experimentally investigates the influence of mechanical proprieties of substrates (counter-faces) when in contact with mushroom-shaped biomimetic adhesive microstructures. The biomimetic mushroom-shaped microstructure tape was made of poly(vinylsiloxane) (PVS) and manufactured by Gottlieb Binder GmbH (Holzgerlingen, Germany) [15]. The counter-faces were cast via replication with three different materials i.e., (i) PVS (Poly-vinyl siloxane); (ii) SILFLO©, which is a brand of silicone impression material; and (iii) a hard Epoxy. The adhesive properties under different contact conditions were investigated using a customized test-rig. The results of this work will help us to identify the key mechanical proprieties responsible for the observed variation in pull-off adhesion force. The effects of counter-face mechanical properties on the adhesion of mushroom-shaped biomimetic microstructures were experimentally investigated under different environmental and operational conditions. The following conclusions were drawn:

▪ In smooth and rigid counter-faces tested under dry contact conditions, mushroom-shaped micro-structures generated almost 6 times more adhesion strength than a smooth control reference. This result is in full agreement with other results reported in the literature [20], although different test-rigs and samples shapes were used.
▪ Under dry contact conditions, soft counter-faces led to lower adhesion than hard counter-faces. This different behavior seemed to be related to the change in the interfacial stress distribution [21].
▪ Under wet conditions, soft counter-faces led to higher adhesion than hard counter-faces. This result can be explained by both additional capillary forces due to the formation of liquid bridges and, possibly, more suction effect favored by the elastic deformation of the mushroom cap and counter-face [24].

In summary, the adaptation and proper use of the adhesive capabilities of biomimetic adhesive microstructures can advance studies in the field of adhesion and promote adhesion to soft surfaces in dry and wet environments. An example of a potential application is the field of biomedicine.

## Figures and Tables

**Table 1:** Average values of the main roughness parameters obtained using four measurements at different zones on each tested counter-face.

Surface Material	*Ra* um	*Rq* um	*Rz* um	*Rpk* nm	*Rvk* nm	Wettability Angle
PVS	0.12	0.15	2.22	142.52	201.19	114.5
SILFLO©	0.59	0.85	13.78	1246.42	1022.09	108.05
EPOXY	0.27	0.35	2.95	366.30	474.30	97.8

## Data Availability

Not applicable

## References

[1] Gorb S.N., Varenberg M. (2007). Mushroom-shaped geometry of contact elements in biological adhesive systems. J. Adhes. Sci. Technol..

[2] Parness A., Soto D., Esparza N., Gravish N., Wilkinson M., Autumn K., Cutkosky M. (2009). A microfabricated wedge-shaped adhesive array displaying gecko-like dynamic adhesion, directionality and long lifetime. J. R. Soc. Interface.

[3] Jagota A., Hui C.-Y. (2011). Adhesion, friction, and compliance of bio-mimetic and bio-inspired structured interfaces. Mater. Sci. Eng. R Rep..

[4] Gorb S., Varenberg M., Peressadko A., Tuma J. (2007). Biomimetic mushroom-shaped fibrillar adhesive microstructure. J. R. Soc. Interface.

[5] Aksak B., Murphy M.P., Sitti M. (2007). Adhesion of Biologically Inspired Vertical and Angled Polymer Microfiber Arrays. Langmuir.

[6] Smith A.M., Callow J.A. (2006). Biological Adhesives.

[7] Santos D., Spenko M., Parness A., Kim S., Cutkosky M. (2007). Directional adhesion for climbing: Theoretical and practical considerations. J. Adhes. Sci. Technol..

[8] Carbas R.J.C., da Silva L.F.M., Marques E.A.S., Lopes A.M. (2013). Effect of post-cure on the glass transition temperature and mechanical properties of epoxy adhesives. J. Adhes. Sci. Technol..

[9] Heepe L., Varenberg M., Itovich Y., Gorb S.N. (2011). Suction component in adhesion of mushroom-shaped microstructure. J. R. Soc. Interface.

[10] Murphy M.P., Kim S., Sitti M. (2009). Enhanced Adhesion by Gecko-Inspired Hierarchical Fibrillar Adhesives. ACS Appl. Mater. Interfaces.

[11] Meng F., Liu Q., Wang X., Tan D., Xue L., Barnes W.J.P. (2019). Tree frog adhesion biomimetics: Opportunities for the development of new, smart adhesives that adhere under wet conditions. Philosophical Transactions of the Royal Society A: Mathematical. Phys. Eng. Sci..

[12] Varenberg M., Gorb S. (2008). A beetle-inspired solution for underwater adhesion. J. R. Soc. Interface.

[13] Kovalev A.E., Varenberg M., Gorb S.N. (2012). Wet versus dry adhesion of biomimetic mushroom-shaped microstructures. Soft Matter.

[14] Ranc H., Servais C., Chauvy P.-F., Debaud S., Mischler S. (2006). Effect of surface structure on frictional behaviour of a tongue/palate tribological system. Tribol. Int..

[15] Tuma J. (2005). Patent “Process for creating adhesion elements on a substrate material”.

[16] Peressadko A., Gorb S.N. (2004). When less is more: Experimental evidence for tenacity enhancement by division of contact area. J. Adhes..

[17] Badler D., Kasem H. (2020). Synergetic effect of the simultaneous use of different biomimetic adhesive micro-structures on tribological performances. Biotribology.

[18] Gorb S.N. (2007). Visualisation of Native Surfaces by Two-Step Molding. Microsc. Today.

[19] Kasem H., Cohen Y. (2017). Effect of counterface roughness on the friction of bionic wall-shaped microstructures for gecko-like attachments. Bioinspir. Biomim..

[20] Kasem H., Varenberg M. (2013). Effect of counterface roughness on adhesion of mushroom-shaped microstructure. J. R. Soc. Interface.

[21] Voigt D., Schuppert J.M., Dattinger S., Gorb S.N. (2008). Sexual dimorphism in the attachment ability of the Colorado potato beetle Leptinotarsa decemlineata (Coleoptera: Chrysomelidae) to rough substrates. J. Insect Physiol..

[22] Carbone G., Pierroac E., Gorb S.N. (2011). Origin of the superior adhesive performance of mushroom-shaped microstructured surfaces. Soft Matter.

[23] Afferrante L., Carbone G. (2013). The Mechanisms of Detachment of Mushroom-Shaped Micro-Pillars: From Defect Propagation to Membrane Peeling. Macromol. React. Eng..

[24] Heepe L., Wolff J.O., Gorb S.N. (2016). Influence of ambient humidity on the attachment ability of ladybird beetles (*Coccinella septempunctata*). Beilstein J. Nanotechnol..

[25] Persson B.N.J. (2003). On the mechanism of adhesion in biological systems. J. Chem. Phys..

[26] Kasem H., Tsipenyuk A., Varenberg M. (2015). Biomimetic wall-shaped hierarchical microstructure for gecko-like attachment. Soft Matter.

[27] Badler D., Goltsberg R., Ammar A.A., Kasem H. (2023). Experimental study of adhesion, friction, and peeling of biomimetic combined micro-mushroom and micro-spatulae textures. Tribol. Int..

